# The Cleavage Effect of Mesenchymal Stem Cell and Its Derived Matrix Metalloproteinase‐2 on Extracellular α‐Synuclein Aggregates in Parkinsonian Models

**DOI:** 10.5966/sctm.2016-0111

**Published:** 2016-10-11

**Authors:** Se Hee Oh, Ha Na Kim, Hyun Jung Park, Jin Young Shin, Dong Yeol Kim, Phil Hyu Lee

**Affiliations:** ^1^ Department of Neurology, Yonsei University College of Medicine, Seoul, Republic of Korea; ^2^ Severance Biomedical Science Institute, Yonsei University, Seoul, Republic of Korea; ^3^ Department of Pharmacology, Ajou University School of Medicine, Suwon, Republic of Korea

**Keywords:** Parkinson’s disease, Mesenchymal stem cell, Matrix metalloproteinase‐2, α‐Synuclein, Proteolysis

## Abstract

Ample evidence has suggested that extracellular α‐synuclein aggregates would play key roles in the pathogenesis and progression of Parkinsonian disorders (PDs). In the present study, we investigated whether mesenchymal stem cells (MSCs) and their derived soluble factors could exert neuroprotective effects via proteolysis of extracellular α‐synuclein. When preformed α‐synuclein aggregates were incubated with MSC‐conditioned medium, α‐synuclein aggregates were disassembled, and insoluble and oligomeric forms of α‐synuclein were markedly decreased, thus leading to a significant increase in neuronal viability. In an animal study, MSC or MSC‐conditioned medium treatment decreased the expression of α‐synuclein oligomers and the induction of pathogenic α‐synuclein with an attenuation of apoptotic cell death signaling. Furthermore, we identified that matrix metalloproteinase‐2 (MMP‐2), a soluble factor derived from MSCs, played an important role in the degradation of extracellular α‐synuclein. Our data demonstrated that MSCs and their derived MMP‐2 exert neuroprotective properties through proteolysis of aggregated α‐synuclein in PD‐related microenvironments. Stem Cells Translational Medicine *2017;6:949–961*

**Significance Statement**

Misfolded α‐synuclein can be released from cells and transmitted from one brain area to others through cell‐to‐cell propagation in Parkinson’s disease (PD). Therefore, extracellular α‐synuclein aggregates would play key roles in the pathogenesis and progression of PD. Proteolysis is the protective mechanism by which mesenchymal stem cells (MSCs) exert neuroprotective properties through proteolysis of aggregated α‐synuclein into soluble forms, which is the actual protective mechanism, and matrix metalloproteinase‐2 is one of the MSC‐derived soluble factors that cleaved preformed fibrils of α‐synuclein. The data suggest that the disassembly of α‐synuclein aggregation to control PD‐related microenvironments using MSCs may have a significant impact on future PD treatment strategies.

## Introduction

Parkinson’s disease (PD) is characterized pathologically by the progressive loss of dopaminergic neurons in the substantia nigra region of the midbrain and the presence of Lewy bodies, proteinaceous fibrillar cytoplasmic inclusions that are composed mainly of aggregated α‐synuclein [1]. Since genetic analyses have identified three missense mutations in the α‐synuclein gene (SNCA), as well as duplication and triplication of the locus containing SNCA in familial forms of PD [2, 3], the pathophysiological role of α‐synuclein has been a target of extensive investigations in PD research.

Other than missense mutations in α‐synuclein, many other factors and events have been reported to influence the fibrillization of α‐synuclein, and these can be involved in the formation of α‐synuclein inclusions in sporadic PD. α‐Synuclein consists of 140 amino acids and is divided into the N‐terminal region (amino acid residues 1–60), the central region (amino acids residues 61–95), and the C‐terminal region (amino acid residues 96–140) [4]. Although the precise role of α‐synuclein in normal function is unknown, it exists natively as an unfolded cytoplasmic protein in neuronal synaptic terminals. However, there is evidence that overexpression and misfolding of α‐synuclein directly increases cell death and toxicity in mammalian cells, leading to a decrease in neurite outgrowth and cell adhesion due to disrupted degradation systems [5]. The α‐synuclein aggregates of monomeric form, oligomeric intermediate, or fibrillar form are thought to be a critical step in the pathogenesis of PD as well as other α‐synucleinopathies of multiple‐system atrophy and dementia with Lewy bodies, although the toxicity seems to differ depending on the aggregate form [6]. Thus, similar to other proteinopathies in the central nervous system, α‐synucleinopathies share key molecular characteristics of toxicity of protein aggregates.

Both oligomeric and monomeric α‐synuclein have been detected in cerebrospinal fluid and plasma samples from PD patients, suggesting that small aggregates of α‐synuclein access the extracellular space [7, 8, 9]. Moreover, previous animal and clinical data have suggested that misfolded α‐synuclein can be released from cells by exocytosis and could then be transmitted from one brain area into others through cell‐to‐cell propagation [10, 11]. Experimental evidence showed that an aggregated form of extracellular α‐synuclein can induce neuroinflammation‐mediated neurotoxic signaling through microglial activation and release of proinflammatory factors [12, 13]. Accordingly, removal of aggregated α‐synuclein from the extracellular space is important for neuronal survival, and, thus, strategies targeting modulation of aggregated α‐synuclein in the extracellular space may be future therapeutic options in patients with PD.

Mesenchymal stem cells (MSCs) are multipotent stem cells that reside in many adult tissues, such as adult bone marrow, liver, muscle connective tissue, amniotic fluid, placenta, umbilical cord blood, or dental pulp, that are capable of differentiating into various cell types under appropriate conditions. Additionally, MSCs secrete various cytotropic factors, including neurotrophic growth factors, chemokines, cytokines, and extracellular matrix protein, which, in turn, exert neuroprotective effects [14, 15, 16, 17]. Our previous studies showed that MSCs have potent neuroprotective effects in animal models of PD through modulation of neuroinflammation, inhibition of apoptotic cell death, increases in neurogenesis and neuronal differentiation, inhibition of cell‐to‐cell transmission of extracellular α‐synuclein, and enhancement of autophagy [18, 19, 20, 21, 22, 23, 24]. In the present study, we evaluated whether MSCs would degrade aggregated α‐synuclein and thus exert a neuroprotective effect through modulation of proteolytic cleavage of α‐synuclein, using in vitro and in vivo models of PD. Furthermore, we determined that matrix metalloproteinase‐2 (MMP‐2), one of the biological molecules secreted from MSCs, plays a crucial role in the modulation of aggregated α‐synuclein.

## Materials and Methods

### α‐Synuclein Aggregate Preparation and Fluorescent Dye Labeling

Recombinant α‐synuclein (200 μM in phosphate‐buffered saline [PBS]) was agitated at 37°C (250 rpm) for 14 days. After brief sonication, the protein was incubated for another 7 days. Aggregated protein was collected by ultracentrifugation at 200,000*g* for 1 hour, and the pellet was resuspended in PBS with brief sonication. Alexa Fluor 488 labeling of α‐synuclein aggregate was performed according to the manufacturer’s instructions (Thermo Fisher Scientific Life Sciences, Oakwood Village, OH, https://www.thermofisher.com). Briefly, aggregate proteins were incubated with 24‐fold molar excess of Alexa Fluor 488 at room temperature for 1 hour. Excess unbound Alexa Fluor 488 dye was removed by passing through a desalting column.

### MSCs and SH‐SY5Y Culture

Frozen vials of characterized human MSCs at passage 2 were obtained from the Severance Hospital Cell Therapy Center (Seoul, South Korea; Institutional Review Board no. 4‐2008‐0643). Human neuroblastoma cell line SH‐SY5Y cells were obtained from the Korean Cell Line Bank (Seoul, South Korea). Both MSCs and SH‐SY5Y cells were maintained in Dulbecco’s modified Eagle’s medium (DMEM, GE Healthcare Life Sciences, Piscataway, NJ, http://www.gelifesciences.com) supplemented with 10% fetal bovine serum (FBS, GE Healthcare) and an antibiotic mixture of penicillin and streptomycin (1%, GE Healthcare). When these cells reached 70%–80% confluence, they were trypsinized and subcultured. These cells were cultivated in a humidified incubator at 37°C and 5% CO_2_ before use. For in vitro experiments, SH‐SY5Y was plated at a density (of 1.5 × 10^4^ cells per cm^2^) and treated with conditioned medium (CM).

### Preparation of Cell CM and Materials

CM was obtained from MSCs at passage 5 and SH‐SY5Y cells. To collect the CM, MSCs and SH‐SY5Y cells were first cultured in FBS‐DMEM, 100 U/ml penicillin, and 100 µg/ml streptomycin and then incubated at 37°C in a humid atmosphere with 5% CO_2_. When the cells reached 70%–80% confluence, they were washed three times with 1× PBS incubated in serum‐free medium (OPTIMEM, Thermo Fisher) at 37°C in a humid atmosphere with 5% CO_2_ for 72 hours. The medium was collected and cleared by 10‐minute centrifugation at 1,200*g*; protein concentration was adjusted with OPTIMEM to 200 µg/ml and sterilized by filtration through a 220‐nm syringe filter (Corning, Corning, NY, http://www.corning.com) and stored at −20°C until used. The media of MSCs and SH‐SY5Y cells were both assumed to contain various paracrine molecules. For in vitro experiments, recombinant human MMP‐2 protein was obtained from Neuromics (Edina, MN, http://www.neuromics.com) (supplemental online Fig. 1). The purified α‐synuclein fibrils (10 µM) were incubated with MMP‐2 (2.2 µg; 1:0.02 substrate/enzyme molar ratio) in an MMP‐reaction buffer (20 mM Tris‐HCl, pH 7.5, 150 mM NaCl, 5 mM CaCl_2_, and 0.5 mM ZnCl_2_) and with CM in a humidified incubator at 37°C for 3 days, and the reaction products were subjected to sodium dodecyl sulfate polyacrylamide gel electrophoresis (SDS‐PAGE) and structural analysis. Reaction mixtures were then centrifuged at 65,000 rpm for 1 hour, supernatants were saved (soluble fraction), and pellets (insoluble fraction) were washed once with PBS. For in vivo injection, CM from 1 × 10^6^ cells was centrifuged to remove cell debris and concentrated by using VIVASPIN6 centrifugal filter devices (Sartorius Stedim, Goettingen, Germany, https://www.sartorius.com) at 4°C for 1 hour.

### Two‐Dimensional PAGE

Two‐dimensional polyacrylamide gel electrophoresis (2D‐PAGE) was basically performed as described previously [25]. Briefly, 200 μg of protein extract was separated by isoelectric focusing by using an immobilized pH gradient strip with a nonlinear pH gradient of 4–10 (Genomine, Pohang, Korea, http://www.genomine.co.kr) for the first dimension and then SDS‐PAGE (26 × 20‐cm format) for the second dimension. Proteins were detected by alkaline silver staining as described previously [26]. Image analysis and quantification of protein spots were performed by using the PDQuest software (Bio‐Rad, Hercules, CA, http://www.bio‐rad.com). The quantity of protein in each spot was normalized relative to the total valid spot intensity.

### Protein Identification by Mass Spectrometry

For protein identification by peptide mass fingerprinting (PMF), protein spots were excised, digested with trypsin (Promega, Madison, WI, http://www.promega.com), mixed with α‐cyano‐4‐hydroxycinnamic acid in 50% acetonitrile/0.1% trifluoroacetic acid, and subjected to matrix‐assisted laser desorption ionization‐time of flight mass spectrometry (MALDI‐TOF/MS) analysis by Microflex LRF 20 (Bruker Daltonics, Seoul, South Korea, https://www.bruker.com) as described [27]. Spectra were collected from 300 shots per spectrum over the *m*/*z* range 600–3,000 and calibrated by two‐point internal calibration using trypsin autodigestion peaks (*m*/*z* 842.5099, 2,211.1046). The peak list was generated by using Flex Analysis 3.0 (Bruker Daltonics). The thresholds used for peak‐picking were as follows: 5,000 for minimum resolution of monoisotopic mass, 2.5 for the signal‐to‐noise ratio. The profound (http://prowl.rockefeller.edu/prowl‐cgi/profound.exe) program was used to search the human National Center for Biotechnology Information Nonredundant database for protein identification. The following parameters were used for the database search: trypsin as the cleaving enzyme, a maximum of one missed cleavage, iodoacetamide as a complete modification, oxidation as a partial modification, monoisotopic masses, and a mass tolerance of ±0.1 Da. PMF acceptance criteria were based on probability scoring.

### Thioflavin Fluorescence Assay

A stock solution of 100 μM Thioflavin T (ThT, Sigma‐Aldrich, St. Louis, MO, http://www.sigmaaldrich.com) was prepared and filtered through a 0.2‐μm polyether sulfone filter (Pall Corp., Port Washington, NY, http://www.pall.com). To each well was added 40 μl of α‐synuclein with CM and 50 μl of ThT (10 μM) in 100 mM glycine‐NaOH (pH 8.5). A black 96‐well microplate (SPL Life Sciences Co., Pocheon, South Korea, http://spllabware.en.ec21.com) was centrifuged briefly at 500 rpm to remove air pockets and equilibrated for 5 min. Fluorescence was measured with a Varioskan Flash (Thermo Fisher) with excitation at 450 nm (bandwidth, 10 nm) and emission at 482 nm (bandwidth, 10 nm).

### Electron Microscopy

Samples were prepared from 5‐μl aliquots of relevant aggregation, and reaction was absorbed onto a glow‐discharged carbon support film, washed twice using 100 μl of distilled water, and negatively stained with 2% (wt/vol) uranyl acetate. Grids were examined at ×100,000 to ×150,000 magnification, and images were examined with a JEM‐100CX electron microscope (JEOL, Tokyo, Japan, www.jeol.co.jp) at 80 kV.

### Cell Viability Analysis

SH‐SY5Y cells were harvested and plated in 96‐well polystyrene plates (Corning) at a concentration of 1.5 × 10^4^ cells per 100 μl of medium per well. Plates were incubated at 37°C for 24 hours to allow cells to attach. After 24 hours, the medium was exchanged with 100 μl of the preincubated mixtures of α‐synuclein with CM. The same volume of DMEM was added to the control cultures. Plates were then incubated at 37°C for an additional 24 and 48 hours. Cell viability was measured by 3‐[4,5‐dimethylthiazol‐2‐yl]‐2,5‐diphenyltetrazolium bromide (MTT) reduction assays essentially as described [28]. Briefly, after the cells were incubated with the various medium samples, MTT was added to a final concentration of 0.5 mg/ml. After incubation at 37°C for 3 hours, the plates were centrifuged, and the medium was aspirated from each well. The absorbance was measured by an enzyme‐linked immunosorbent assay (ELISA) microplate reader (VersaMax, Molecular Devices, Sunnyvale, CA, http://www.moleculardevices.com) at 490 nm. Cell viability was calculated by dividing the absorbance of wells containing samples (corrected for background) by the absorbance of wells containing medium alone (corrected for background).

### Reverse‐Transcription Polymerase Chain Reaction

To knock down MMP‐2 in MSCs, an MMP‐2 small interfering RNA (siRNA) construct (Santa Cruz Biotechnology, Santa Cruz, CA, http://www.scbt.com) was purchased and tested for knockdown efficiency. MSCs were plated at a density of 1 × 10^5^ per cm^2^ and incubated with 8 μl of 20 μM siRNA (final concentration 40 nM) in 100 μl of OPTIMEM (Thermo Fisher) containing 10 μl of Lipofectamine 2000 (Thermo Fisher). After 72 hours, total RNA was extracted from the MSCs by using TRIzol reagent (Thermo Fisher) in accordance with the manufacturer’s protocol. An equal amount of RNA (1 µg) in each experiment was reverse‐transcribed by using amfiRivert cDNA Synthesis Premix (GenDEPOT, Barker, TX, http://www.gendepot.com). Subsequently, 2 µl of cDNA were used as a template for reverse‐transcription polymerase chain reaction (RT‐PCR) analysis in and amfiRivert 1‐Step RT‐PCR Kit (GenDEPOT). PCR was performed by using 10 pmol of primers for human MMP‐2 (forward, 5′‐CATACAAAGGGATTGCCAGGA ‐3′; reverse, 5′‐GGTATTGCACTGCCAACTCT‐3′). After an initial denaturation at 95°C for 2 minutes, 30 cycles of PCR were performed, consisting of denaturation (1 minute, 95°C), annealing (1 minute, 54°C), and extension (1 minute, 72°C), followed by a final extension step (5 minutes, 72°C). The PCR products were separated by electrophoresis on 2% agarose gels (iNtRON Biotechnology, Seongnam, South Korea, http://intronbio.com) and stained with ethidium bromide (Sigma‐Aldrich). Gels were examined under UV illumination (DNR Bio‐Imaging Systems, Jerusalem, Israel, http://www.dnr‐is.com). Density was measured by using the Image Gauge software (Version 4.0, Fujifilm Advanced Research Laboratories, Ashigarakami, Japan, http://www.fujifilm.com).

### Animal Study

All procedures were performed in accordance with the Laboratory Animals Welfare Act, the Guide for the Care and Use of Laboratory Animals, and the Guidelines and Policies for Rodent Experiment provided by the Institutional Animal Care and Use Committee at the Yonsei University Health System. Male C57BL/6 mice aged 16 weeks (Orient Bio, Seongnam, South Korea, *www.orient.co.kr*) were acclimated in a climate‐controlled room with a constant 12‐hour light/dark cycle (12‐hour lights on) for 1 week before the initiation of drug administration. To evaluate the modulatory effects of MSCs in an 1‐methyl‐4‐phenyl‐1,2,3,6‐tetrahydropyridine (MPTP)‐induced animal model of PD, mice were injected with MPTP (intraperitoneal injection [i.p.), 30 mg/kg, daily for 5 days]. The mice were randomly divided into three groups (*n* = 5 per group): (a) control, (b) MPTP, and (c) MPTP and MSC. Mice in the MPTP group were injected with saline via tail vein at 3 days after final MPTP injection. Mice in the MSC group were subjected to MSCs into the tail vain (1 × 10^6^ cells per 200 μl) at 3 days after final MPTP injection. All mice were sacrificed at 7 days after MSC injection. To create an α‐synuclein‐inoculated model, α‐synuclein (5 μg per mouse) with or without dynasore (80 μM per mouse) was administered via the neocortex and striatum in accordance with the procedure described previously with minor modifications [11]. Briefly, mice were anesthetized with isoflurane (Baxter, Utrecht, The Netherlands, http://www.baxter.nl), and α‐synuclein with or without dynasore were slowly injected bilaterally into the cortex (0.4 mm posterior to bregma, 1.3 mm lateral to midline, and 0.6 mm ventral to the brain surface) and striatum (0.2 mm posterior to bregma, ±2.0 mm lateral to midline, and 2.6 mm ventral to the brain surface) by using a stainless‐steel injection needle (26 gauge) connected to a 10‐μl Hamilton microsyringe (Hamilton, Reno, NV, http://www.hamiltoncompany.com). The needle was left in place for 10 minutes before being withdrawn slowly. Then, to evaluate whether MSCs disassemble extracellular α‐synuclein aggregates, the mice were randomly divided into three groups (*n* = 5 per group): (a) control, (b) α‐synuclein, and (c) α‐synuclein and MSC. Mice in the α‐synuclein group were injected with saline via tail vein at 1 day after α‐synuclein inoculation (postoperative day 1). Mice in the MSC group were subjected to MSCs into the tail vain (1 × 10^6^ cells per 200 μl) on postoperative day 1, and all mice were sacrificed on postoperative day 7 (cortical injection group) or 30 (striatal injection group). Finally, to evaluate the modulatory effects of MSCs on extracellular α‐synuclein aggregates, the mice were randomly divided into three groups (*n* = 5 per group): (a) fresh medium, (b) MSC‐CM, and (c) MMP‐2. Mice in each group were subjected to medium delivery on postoperative day 1, and all mice were sacrificed on postoperative day 4.

### Brain Sample Preparation

For immunochemical analysis, all mice were deeply anesthetized with chloral hydrate (I.P., 0.4 g/kg; Sigma‐Aldrich) and then perfused with 4% paraformaldehyde (Sigma‐Aldrich) in 0.1 M phosphate buffer (pH 7.4). The brains were embedded in paraffin, and coronal sections 4‐µm thick were then cut and placed on slides.

### Immunocytochemistry and Immunohistochemistry

SH‐SY5Y cells and brain sections were washed twice in PBS and incubated in 0.2% Triton X‐100 (Sigma‐Aldrich) for 30 minutes at room temperature. They were blocked with 0.5% bovine serum albumin (BSA; Sigma‐Aldrich) for 30 minutes. After blocking, they were rinsed three times with 0.5% BSA and incubated overnight at 4°C with specific primary antibodies. The primary antibodies used were as follows: mouse anti‐α‐synuclein (EMD Millipore, Billerica, MA, http://www.emdmillipore.com), rabbit anti‐α‐synuclein (phospho S129, Abcam, Cambridge, MA, http://www.abcam.com), rabbit anti‐MMP‐2 (Abcam), mouse anti‐tyrosine hydroxylase antibody (TH, Sigma‐Aldrich), and mouse anti‐nuclear mitotic apparatus protein antibody (NuMA, EMD Millipore). Immunofluorescence labeling was carried out by incubating the cells with rabbit anti‐IgG Alexa Fluor 555 (Thermo Fisher Scientific Life Sciences), mouse anti‐IgG Cy‐3 (EMD Millipore), rabbit anti‐IgG Cy‐3 (EMD Millipore), and rabbit anti‐IgG FITC (EMD Millipore). The cell nuclei were counterstained with 4′,6‐diamidino‐2‐phenylindole (Thermo Fisher). The TH antibodies were detected with 0.05% diaminobenzidine (Vector Laboratories, Burlingame, CA, http://vectorlabs.com). The immunostained cells were analyzed by using bright‐field microscopy and viewed under a Zeiss LSM 700 confocal imaging system (Zeiss, Stuttgart, Germany, http://www.zeiss.com). To analyze the localizations of antigens in double‐stained samples, immunofluorescence images were created from the same tissue sections and merged by using the Zeiss ZEN software.

### Western Blotting

Brain tissues were dissolved in ice‐cold RIPA buffer (50 mM Tris‐HCl, pH 8.0, with 150 mM sodium chloride, 1.0% Igepal CA‐630, 0.5% sodium deoxycholate, and 0.1% SDS) (Sigma‐Aldrich) plus protease inhibitor cocktail (Sigma‐Aldrich). The lysates were centrifuged at 4°C for 20 min (14,000*g*), and supernatants were transferred to fresh tubes. For soluble and insoluble fractions, 5 μl of 10% Triton X‐100 was added to 45 μl of protein to produce a 1% solution of Triton X‐100 and vortexed. The extraction was performed for 15 minutes on ice, and the cell extract was centrifuged at 14,000*g* for 10 minutes. The supernatant (soluble fraction) was saved, and the pellet (insoluble fraction) was resuspended in 10 μl of RIPA buffer. Briefly, 50 and 100 μg of protein were separated by SDS‐gel electrophoresis and transferred onto hydrophobic polyvinylidene difluoride membranes (GE Healthcare). The membranes were blocked in nonfat milk (BD, Franklin Lakes, NJ, http://www.bd.com). Membranes were probed with the following primary antibodies: rabbit anti‐α‐synuclein (EMD Millipore), mouse anti‐α‐synuclein (Thermo Fisher), rabbit anti‐α‐synuclein (phospho‐S129, Abcam), rabbit anti‐MMP‐2 (Cell Signaling Technology, Beverly, MA, http://www.cellsignal.com), rabbit anti‐caspase‐3 (Cell Signaling Technology), and rabbit anti‐α‐tubulin (Santa Cruz Biotechnology). As secondary antibodies, a 1:10,000 dilution of horseradish peroxidase (HRP)‐conjugated goat anti‐rabbit antibody (GenDEPOT) and anti‐mouse antibody (GenDEPOT) were used. Antigen‐antibody complexes were visualized with a chemiluminescence system (Santa Cruz Biotechnology), followed by exposure to x‐ray film (Fujifilm Advanced Research Laboratories) and ImageQuant LAS‐4000 (GE Healthcare). For semiquantitative analysis, immunoblotting band densities were measured by computer imaging.

### Measurement of Oligomeric α‐Synuclein

Oligomeric α‐synuclein was measured by using a high‐sensitivity oligomeric α‐synuclein ELISA kit (MyBioSource, San Diego, CA, https://www.mybiosource.com). A total of 50 μl of each diluted sample and standard included in the kit was applied to microtiter plates precoated with antibody that specifically recognized the oligomeric α‐synuclein peptides. The plate was incubated with 100 μl per well of HRP‐conjugate reagent for 1 hour at 37°C. After washing, 350 μl of wash solutions was added to each well, and then invert plate dried by hitting the plate until no moisture appeared. The substrate was added and incubated for 15 minutes at 37°C, and then the reaction was stopped with stop solution. The color reaction was measured with an automatic ELISA microplate reader with the wavelength set at 450 nm. The software (Bio‐Rad) was used to create standard curves and calculate the concentration of the samples.

### Measurement of α‐Synuclein

The amount of α‐synuclein was measured by using a sandwich ELISA kit (AnaSpec, Fremont, CA, http://www.anaspec.com). A total of 10 μl of each diluted sample and standard included in the kit were applied to microtiter plates precoated with antibody that specifically recognized α‐synuclein. After an overnight incubation at 4°C and washing, a detection antibody indirectly linked to an enzyme was applied. After incubation and washing, 350 μl of wash solution was added to each well and then invert plate dried by hitting the plate until no moisture appeared. The substrate was added and incubated for 15 minutes at 37°C, and then the reaction was stopped with stop solution. The color reaction was measured with an automatic ELISA microplate reader (BioTek, Winooski, VT, http://www.biotek.com) with the wavelength set at 450 nm. The software (Bio‐Rad) was used to create standard curves and to calculate the concentration of the samples.

### Stereological Cell Counts

TH‐stained neurons were counted in the right and left substantia nigra pars compacta (SNpc) of every fourth section throughout the entire extent of the SNpc. Each midbrain section was viewed at low power at a random start, and then the number of TH‐stained cells was counted at high power. To avoid double counting of neurons with unusual shapes, TH‐stained cells were counted only when their nuclei were optimally visualized, which occurred only in one focal plane. After all of the TH‐stained neurons were counted, the total numbers of TH‐stained neurons in the SNpc were calculated by using the formula described [29].

### Statistical Analysis

The group means were compared using the Mann‐Whitney *U* test for pairs and the Kruskal‐Wallis analysis for multiple groups. *p* values less than .05 were considered statistically significant. Statistical analyses were performed by using commercially available software (SPSS Version 12.0; IBM, Armonk, NY, http://www.ibm.com).

## Results

### MSC‐CM Disassembles α‐Synuclein Aggregates

**Figure 1:**
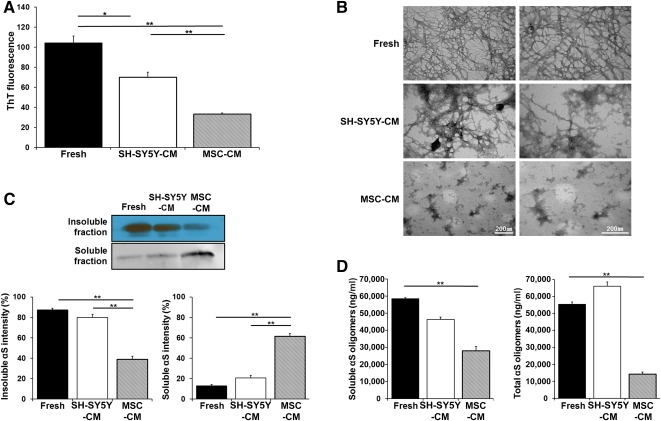
MSCs disassemble α‐synuclein fibril formation. **(A):** Analysis of ThT fluorescence showed that the intensity of αS aggregates was higher in fresh medium, whereas αS aggregates were decreased in the MSC‐CM. **(B):** An electron microscopy analysis showed that αS aggregates incubated in the fresh medium, whereas the amorphous aggregates of nonfibrillar forms were observed in MSC‐CM compared with SH‐SY5Y‐CM. **(C):** The portion of insoluble αS fraction was decreased in MSC‐CM. **(D):** The levels of oligomeric αS in soluble and total fractions were decreased in MSC‐CM compared with fresh medium or SH‐SY5Y‐CM. All data are presented as the means ± SE. ∗, *p* < .05; ∗∗, *p* < .01. Scale bar = 200 nm. Abbreviations: MSC‐CM, conditioned medium obtained from mesenchymal stem cells; αS, α‐synuclein; SH‐SY5Y‐CM, conditioned medium obtained from SH‐SY5Y cells; ThT, Thioflavin T.

To determine whether MSCs can degrade α‐synuclein aggregates, α‐synuclein fibrils were incubated in fresh medium, SH‐SY5Y‐CM, and MSC‐CM. On analysis of thioflavin fluorescence, which is widely used to bind to fibrillar structures, the intensity of α‐synuclein aggregates was markedly higher in fresh medium, whereas α‐synuclein aggregates were significantly decreased in the MSC‐CM compared with fresh or SH‐SY5Y‐CM (Fig. 1A). In addition, electron microscopy (EM) analysis showed that α‐synuclein aggregates incubated in fresh medium exhibited the presence of long fibrils as well as large web‐like networks composed of many fibrils, whereas α‐synuclein aggregates in SH‐SY5Y‐ or MSC‐CM showed a variety of smaller and apparently amorphous aggregates corresponding to nonfibrillar species (Fig. 1B). The amorphous aggregates of nonfibrillar forms were more prominent in MSC‐CM compared with SH‐SY5Y‐CM. Next, we performed Western blot analysis to determine the modulatory effects of MSCs on the soluble and insoluble fractions of α‐synuclein. α‐Synuclein aggregates incubated with fresh medium or SH‐SY5Y‐CM were composed mainly of the insoluble fraction of α‐synuclein, whereas the portion of insoluble α‐synuclein fraction was markedly decreased in MSC‐CM compared with fresh or SH‐SY5Y‐CM (Fig. 1C). In addition, the levels of α‐synuclein oligomers in soluble and total fractions were significantly decreased in MSC‐CM compared with fresh medium or SH‐SY5Y‐CM (Fig. 1D).

### MSC‐Mediated α‐Synuclein Cleavage Increases Cellular Viability

**Figure 2:**
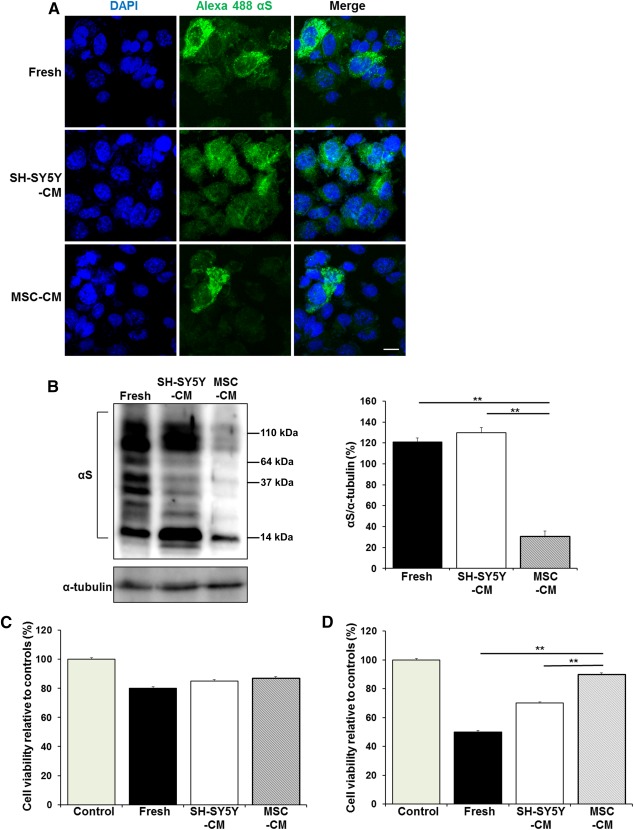
MSCs significantly increase cellular viability. **(A):** Immunofluorescence analysis revealed that the majority of Alexa 488‐labeled αS fibrils were internalized into intracellular cytoplasm in fresh medium, SH‐SY5Y‐CM, and MSC‐CM. **(B):** Western blotting showed that the expression of αS was reduced in MSC‐CM. **(C, D):** Cell viability among the groups of each medium after 24 hour **(C)** or 48 hour **(D)** treatment. All data are presented as the means ± SE. ∗, *p* < .05; ∗∗, *p* < .01. Scale bar = 20 μm. Abbreviations: DAPI, 4′,6‐diamidino‐2‐phenylindole; MSC‐CM, conditioned medium obtained from mesenchymal stem cells; αS, α‐synuclein; SH‐SY5Y‐CM, conditioned medium obtained from SH‐SY5Y cells.

To visualize the endocytosis of α‐synuclein, we used Alexa 488 fluorescently labeled α‐synuclein fibrils. Alexa 488‐labeled α‐synuclein fibrils were incubated with fresh medium, SH‐SY5Y‐CM, or MSC‐CM, and then SH‐SY5Y cells were treated with each medium for 72 hours. Immunofluorescence analysis indicated that the majority of Alexa 488‐labeled α‐synuclein fibrils were rapidly internalized into the cytoplasm in fresh medium and SH‐SY5Y‐CM, whereas intracellular uptake of Alexa 488‐labeled α‐synuclein was markedly decreased in MSC‐CM (Fig. 2A). In addition, Western blot analysis showed that the amount of α‐synuclein in SH‐SY5Y cells was significantly lower in MSC‐CM compared with fresh medium or SH‐SY5Y‐CM, further confirming that MSC‐CM significantly blocked endocytosis of extracellular α‐synuclein (Fig. 2B). Next, to evaluate the cytotoxic consequences of α‐synuclein internalization, we compared neuronal viability of SH‐SY5Y cells treated with fresh medium, SH‐SY5Y‐CM, or MSC‐CM for 24 and 48 hours by MTT assay. When SH‐SY5Y cells were treated with each medium for 24 hours, neuronal viability did not differ significantly among the groups (Fig. 2C). However, SH‐SY5Y cell viability was significantly increased in MSC‐CM after 48 hours of treatment compared with fresh medium and SH‐SY5Y‐CM (Fig. 2D).

### α‐Synuclein Fibrils Are Cleaved by the MSC‐Derived Protein MMP‐2

**Figure 3:**
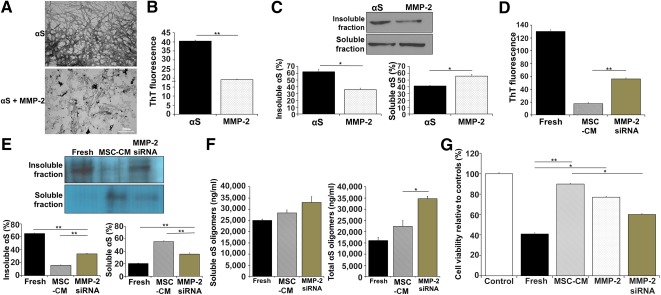
Mesenchymal stem cell‐derived MMP‐2 accelerates degradation of α‐synuclein fibrils. **(A):** An electron microscopy analysis showed that α‐synuclein (αS) aggregates in the presence of MMP‐2 exhibited a large portion of smaller and apparently amorphous aggregates. **(B):** On the thioflavin fluorescence assay, αS aggregates in the presence of MMP‐2 were decreased compared with αS aggregates alone. **(C):** Western blotting revealed that MMP‐2 treatment led to a decrease in the portion of insoluble αS fraction. **(D–F):** When αS fibrils were incubated with MMP‐2 siRNA‐treated MSC‐CM, the intensity of αS aggregates was increased relative to MSC‐CM. MMP‐2 siRNA‐treated medium increased the expression of αS insoluble fractions and oligomeric αS levels compared with MSC‐CM or MMP‐2 treatment . **(G):** SH‐SY5Y cell viability that was increased in MSC‐CM relative to fresh medium was attenuated in the presence of MMP‐2 siRNA‐treated medium. All data are presented as the means ± SE. ∗, *p* < .05; ∗∗, *p* < .01. Scale bar = 200 nm. Abbreviations: MMP‐2, matrix metalloproteinase‐2; MSC‐CM, conditioned medium obtained from mesenchymal stem cells; αS, α‐synuclein; siRNA, small interfering RNA; ThT, Thioflavin T.

To determine proteins secreted from MSCs, we collected three independent samples of fresh medium, SH‐SY5Y‐CM, and MSC‐CM for 2D‐PAGE and MALDI‐TOF/MS proteomics. On the 2D‐PAGE gel of total protein extracted from each medium at 72 hours, spot intensities and patterns were largely similar between independent samples from the SH‐SY5Y‐CM and MSC‐CM groups (supplemental online Fig. 2). MMP‐2 was expressed in both MSCs and SH‐SY5Y cells; however, the expression level of MMP‐2 was much higher in MSCs relative to SH‐SY5Y cells (supplemental online Fig. 3). α‐Synuclein fibrils were incubated with MMP‐2 for 72 hours to examine their effects on α‐synuclein degradation. EM analysis showed that α‐synuclein aggregates in the presence of MMP‐2 exhibited a large portion of smaller and apparently amorphous aggregates compared with the absence of MMP‐2 (Fig. 3A). In a ThT fluorescence assay, α‐synuclein aggregates that exhibited β‐sheet formation in the presence of MMP‐2 were significantly decreased compared with those in the absence of MMP‐2 (Fig. 3B). Consistent with these results, Western blot analysis indicated that MMP‐2 treatment led to a marked decrease in the insoluble α‐synuclein fraction from α‐synuclein aggregates, which were composed mainly of the insoluble fraction (Fig. 3C). Next, to confirm that MMP‐2 had a cleavage effect on α‐synuclein aggregates, we used an MMP‐2 siRNA construct to knock down MMP‐2 expression in MSCs. As expected, MMP‐2 expression was markedly decreased in MMP‐2 siRNA‐treated MSCs (supplemental online Fig. 4). The ThT fluorescence assay indicated that when α‐synuclein fibrils were incubated with CM of MMP‐2 siRNA‐treated MSCs, the intensity of α‐synuclein aggregates was significantly increased relative to incubation in MSC‐CM (Fig. 3D). Western blot analysis revealed that MMP‐2 siRNA‐treated medium increased the level of the α‐synuclein insoluble fraction with a concomitant reduction in the soluble fraction compared with MSC‐CM, suggesting that MMP‐2 could markedly enhance α‐synuclein cleavage (Fig. 3E). In addition, MMP‐2 siRNA‐treated medium significantly increased oligomeric α‐synuclein levels in soluble and total fractions compared with MSC‐CM or MMP‐2 treatment (Fig. 3F). Finally, to evaluate the effects of MSCs on neuronal viability after knocking down of MMP‐2 expression, SH‐SY5Y cells were treated with CM of MMP‐2 siRNA‐treated MSCs that had been preincubated with α‐synuclein fibrils. SH‐SY5Y cell viability that was significantly increased in MSC‐CM relative to fresh medium was prominently attenuated in the presence of MMP‐2 siRNA‐treated MSC‐CM (Fig. 3G). These results suggested that the MSC‐derived factor, MMP‐2, likely accelerated degradation of α‐synuclein fibrils. However, the intensity of α‐synuclein aggregates, the proportion of the insoluble fraction, and neuronal viability in MMP‐2 siRNA‐treated MSC‐CM were not comparable to the corresponding values in fresh medium or SH‐SY5Y‐CM (Fig. 3D–3G), suggesting that other soluble factors in addition to MMP‐2 may be involved in α‐synuclein aggregate modulation.

### MSCs Express MMP‐2 and Could Modulate α‐Synuclein Expression in MPTP‐Treated Parkinsonian Animals

**Figure 4:**
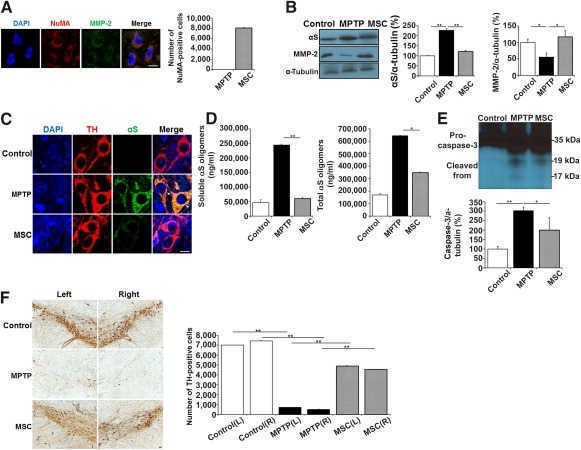
MSCs modulate α‐synuclein in MPTP‐treated Parkinsonian animals through induction of MMP‐2. **(A):** Immunohistochemical analysis revealed that NuMA‐positive cells were coimmunostained with MMP‐2 in the MSC‐treated animals. **(B):** MSC administration decreased αS expression with a restoration of MMP‐2 expression in MPTP‐treated animals. **(C):** MSC administration in MPTP‐treated animals decreased the immunoreactivity of αS with accumulation of aggregated αS (white arrows) in the TH‐positive neurons. **(D):** MSC administration in MPTP‐treated animals decreased the levels of soluble and total αS oligomers in the midbrain compared with only MPTP‐treated animals. **(E, F):** MSC treatment decreased the expression of cleaved caspase‐3 fragment and increased the survival of TH‐positive neurons compared with only MPTP‐treated animals. All data are presented as the means ± SE. ∗, *p* < .05; ∗∗, *p* < .01. Scale bars = 10 μm. Abbreviations: DAPI, 4′,6‐diamidino‐2‐phenylindole; L, left substantia nigra pars compacta; MMP‐2, matrix metalloproteinase‐2; MPTP, 1‐methyl‐4‐phenyl‐1,2,3,6‐tetrahydropyridine; MSC, mesenchymal stem cell; NuMA, anti‐nuclear mitotic apparatus protein; R, right substantia nigra pars compacta; αS, α‐synuclein; TH, anti‐tyrosine hydroxylase.

To evaluate whether MSCs could modulate MMP‐2 expression in a MPTP‐induced animal model of PD, we attempted to identify MSCs in the midbrain using human‐specific NuMA antibody. Immunohistochemical analysis revealed that NuMA‐positive cells were not detected in only MPTP‐treated animals, whereas approximately 0.9% of injected MSCs that were coimmunostained with MMP‐2 were observed in the MSC treatment group (Fig. 4A). The expression of MMP‐2 in the midbrain was significantly decreased in MPTP‐treated animals compared with controls; however, MSC administration markedly restored MMP‐2 expression in MPTP‐treated animals (Fig. 4B). In addition, MPTP treatment significantly increased the immunoreactivity of α‐synuclein with accumulation of aggregated α‐synuclein in the TH‐positive neurons and the overall level of total α‐synuclein and α‐synuclein oligomers in the midbrain (Fig. 4B–4D). However, MSC administration in MPTP‐treated animals significantly attenuated accumulation of aggregated α‐synuclein and the expression of total α‐synuclein and the levels of α‐synuclein oligomers in the midbrain compared with MPTP‐treated animals (Fig. 4B–4D). Moreover, MSC treatment in MPTP‐treated animals significantly decreased the expression of cleaved caspase‐3 fragment and increased survival of dopaminergic neurons in the midbrain compared with only MPTP‐treated animals (Fig. 4E, 4F).

### MSCs Disassemble Extracellular α‐Synuclein Aggregates via Increased MMP‐2 Expression in α‐Synuclein‐Inoculated Animals

**Figure 5:**
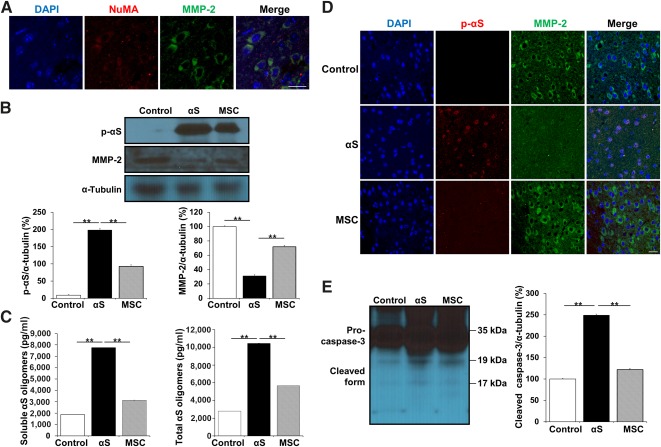
MSC treatment attenuates extracellular α‐synuclein aggregates in α‐synuclein‐inoculated animals. **(A):** Immunohistochemical analysis revealed that NuMA‐positive cells were coimmunostained with MMP‐2 in the MSC‐treated animals. **(B):** MSC treatment decreased the expression of phosphorylated αS and restored MMP‐2 expression in α*S*‐inoculated animals. **(C):** MSC administration decreased the levels of soluble and total αS oligomers in the cortex compared with only α*S*‐inoculated animals. **(D):** MSC treatment in α*S*‐inoculated animals decreased the immunoreactivity of phosphorylated αS and increased the immunoreactivity of MMP‐2. **(E):** MSC treatment decreased the expression of cleaved caspase‐3 fragment compared with only αS‐inoculated animals. All data are presented as the means ± SE. ∗∗, *p* < .01. Scale bars = 10 μm. Abbreviations: DAPI, 4′,6‐diamidino‐2‐phenylindole; MMP‐2, matrix metalloproteinase‐2; MSC, mesenchymal stem cell; NuMA, anti‐nuclear mitotic apparatus protein; p‐αS, phosphorylated α‐synuclein; αS, α‐synuclein.

In α‐synuclein‐inoculated animals, NuMA‐positive cells were coimmunostained with MMP‐2 in the MSC‐treated animals (Fig. 5A), and the expression of MMP‐2 in the cortex was significantly increased in the MSC‐treated animals relative to only α‐synuclein‐inoculated animals (Fig. 5B). Additionally, MSC administration in α‐synuclein‐inoculated animals significantly attenuated the amount of phosphorylated α‐synuclein and the levels of total and soluble α‐synuclein oligomers in the cortex compared with α‐synuclein‐inoculated animals (Fig. 5B, 5C). Moreover, MSC treatment in the α‐synuclein‐inoculated brain significantly attenuated phosphorylated α‐synuclein immunoreactivity with an increase in MMP‐2 immunoreactivity (Fig. 5D), suggesting that MSCs and MSC‐derived MMP‐2 inhibit the induction of pathogenic α‐synuclein by disassembling exogenous α‐synuclein aggregates. Consequently, MSC treatment in α‐synuclein‐inoculated animals significantly decreased the expression of cleaved caspase‐3 fragment in the cortex, compared with only α‐synuclein‐inoculated animals (Fig. 5E). We further examined the effects of MSCs on proteolysis of α‐synuclein aggregates after stereotaxic inoculation of α‐synuclein fibrils into the striatum. Consistent with previous findings, MSC treatment in α‐synuclein‐inoculated animals markedly increased MMP‐2 expression in the striatum, which induced attenuation of the amount of phosphorylated α‐synuclein and α‐synuclein oligomers with decreased caspase‐3 expression (supplemental online Fig. 5).

### α‐Synuclein Aggregate Formation Is Decreased by MSC‐CM and MMP‐2 in α‐Synuclein‐Inoculated Animals

**Figure 6:**
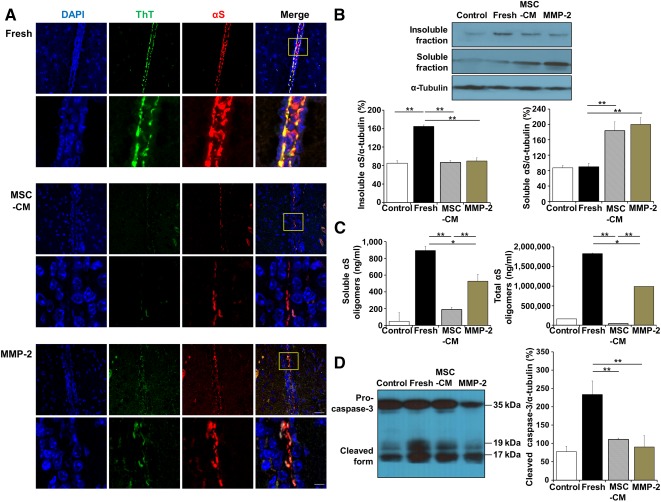
MSC‐CM and MMP‐2 attenuate extracellular α‐synuclein aggregates in α‐synuclein‐inoculated animals. **(A):** Both αS and ThT immunoreactivities in fresh medium‐treated animals were prominent and extended beyond the injection site, whereas these immunoreactivities were localized to the injection site in animals administered MSC‐CM and MMP‐2. The area shown in the yellow box in the upper right is enlarged in lower. **(B, C):** The levels of insoluble and oligomeric αS were decreased in animals receiving MSC‐CM or MMP‐2 compared with fresh medium‐treated animals. **(D):** The cleaved caspase‐3 was clearly observed in animals receiving fresh medium compared with animals receiving MSC‐CM or MMP‐2. All data are presented as the means ± SE. ∗, *p* < .05; ∗∗, *p* < .01. Scale bars = 50 and 10 μm. Abbreviations: DAPI, 4′,6‐diamidino‐2‐phenylindole; MMP‐2, matrix metalloproteinase‐2; MSC‐CM, conditioned medium obtained from mesenchymal stem cells; αS, α‐synuclein; ThT, Thioflavin T.

To elucidate the modulatory effects of MSCs and MMP‐2 on extracellular α‐synuclein aggregates, an endocytosis inhibitor (dynasore) and synthetic α‐synuclein were coinjected into the neocortex to avoid intracellular internalization as much as possible. After 24 hours, fresh medium, MSC‐CM, and MMP‐2 were injected into the same site, and α‐synuclein aggregates were assessed around the injection site 72 hours later. Immunohistochemical analysis showed that both α‐synuclein and ThT immunoreactivities in fresh‐medium‐treated animals were prominent and extended beyond the injection site. However, animals administered MSC‐CM showed markedly reduced levels of immunoreactivity for both α‐synuclein and ThT, and the immunoreactivity was localized to the injection site, indicating that MSC‐CM could attenuate extracellular α‐synuclein aggregate formation. Similar to MSC‐CM, MMP‐2 administration exhibited a modulation of extracellular α‐synuclein aggregates, with deceased and localized immunoreactivity for aggregated α‐synuclein (Fig. 6A). On Western blotting, the proportion of insoluble α‐synuclein fraction relative to the soluble form was increased in fresh medium‐treated animals, whereas the insoluble α‐synuclein fraction was significantly decreased in animals receiving MSC‐CM or MMP‐2 (Fig. 6B). Western blot analysis using human‐specific α‐synuclein antibody also revealed quite similar patterns of inoculated α‐synuclein amounts after MSC‐CM or MMP‐2 treatment (supplemental online Fig. 6). The levels of α‐synuclein oligomers in soluble and total fractions were lower in animals receiving MSC‐CM or MMP‐2 than fresh medium‐treated animals. These α‐synuclein oligomer levels were much lower in MSC‐treated animals relative to MMP‐2‐treated animals (Fig. 6C). Additionally, the cleavage of procaspase‐3 into the active subunits was clearly observed in animals receiving fresh medium compared with animals receiving MSC‐CM or MMP‐2 (Fig. 6D).

## Discussion

The present study demonstrated that MSCs degraded the aggregated form of α‐synuclein both in cellular and animal models of enriched α‐synuclein and exerted a prosurvival effect on neurons through proteolysis of aggregated α‐synuclein. In addition, we showed that MMP‐2, a factor derived from MSCs, plays an important role in the disassembly of aggregated α‐synuclein in these models. Our data suggest that the cleavage effect of MSC‐derived soluble factors is responsible for attenuation of extracellular α‐synuclein aggregates, which may be applicable to future clinical strategies for treatment of PD patients.

It is well known that α‐synuclein has a tendency to aggregate and accumulate, thus forming small intracellular aggregates, which would lead to an increase in cellular toxicity and cell death in various types of α‐synucleinopathies [30, 31, 32]. More importantly, recent studies have provided convincing evidence for cell‐to‐cell propagation of α‐synuclein, showing that α‐synuclein and its aggregates are released from neuronal cells via exocytosis [33, 34], and neurons and glial cells have the ability to take up extracellular α‐synuclein aggregates through endocytosis [35, 36]. In terms of their prion‐like behavior, extracellular α‐synuclein aggregates seem to play key roles in the pathogenesis and progression of PD, and treatment strategies focused on modulation of extracellular α‐synuclein transmission would be clinically relevant for PD. In this regard, the results of immunotherapy targeting aggregated α‐synuclein in animal models of Lewy body diseases are very suggestive, because antibodies against α‐synuclein can modulate aggregated α‐synuclein at several steps of accumulation and propagation [37, 38]. Specifically, antibodies can recognize α‐synuclein oligomers accumulating in the neuronal membrane and, after internalization, enhance degradation of α‐synuclein via autophagy [39, 40]. Furthermore, during extracellular propagation α‐synuclein, circulating antibodies enhance removal of extracellular α‐synuclein aggregates and thus modulate their propagation [38, 41]. In addition, several experimental studies showed that rifampicin, a common antibiotic used in the treatment of tuberculosis and leprosy, has the ability to inhibit α‐synuclein aggregation and disaggregate preformed fibrils and reduce α‐synuclein aggregation in α‐synuclein transgenic mice with decreased neurodegeneration [42, 43]. Therefore, a therapeutic strategy to enhance removal of extracellular α‐synuclein aggregates may be an important pharmacological target in disease‐modifying treatment strategies for PD.

Several studies have demonstrated that MSCs exert neuroprotective effects by secretion of neurotropic molecules that directly or indirectly can modulate neurodegenerative microenvironment [44, 45]. Additionally, MSCs are known to secrete several molecules into the neural niche microenvironment, which could promote endogenous neural repair [46, 47]. Moreover, MSCs have the potential to facilitate proteolysis against the assembled protein structures [48]. In the present study, we found that MSCs have the ability to disaggregate preformed fibrils of α‐synuclein and thus lead to a decrease in level of the insoluble form of α‐synuclein, which was accompanied by increased neuronal survivals. In the in vitro experiments, preformed α‐synuclein aggregates incubated with MSC‐CM showed marked disassembly, and the ratio of insoluble and soluble forms of α‐synuclein was decreased compared with those incubated with fresh medium or SH‐SY5Y‐CM. Interestingly, the present study showed that remnant α‐synuclein in the medium could be internalized into neurons when cocultured with neuronal cells. As expected, the amounts of α‐synuclein aggregates located in the neuronal cytoplasm were significantly decreased in MSC‐CM relative to fresh medium or SH‐SY5Y‐CM, which led to a significant increase in neuronal viability. The modulatory effect of MSCs on α‐synuclein aggregates was further supported by in vivo experiments. MSC treatment in a neurotoxin‐induced animal model of PD significantly attenuated the expression of total and oligomeric forms of α‐synuclein in the midbrain compared with neurotoxin treatment alone, which led to decreased caspase‐3 expression and dopaminergic neuronal loss. Moreover, in α‐synuclein‐inoculated animals, MSCs inhibited the induction of pathogenic α‐synuclein in cortical neurons by disassembling exogenous α‐synuclein aggregates and, consequently, attenuated apoptotic cell death signaling. In addition, administration of MSC‐CM markedly decreased the extent of inoculated α‐synuclein aggregates and significantly decreased expression of the insoluble and oligomeric forms of α‐synuclein in animals treated with enriched α‐synuclein compared with fresh medium. Accordingly, the present study provided additional evidence that MSCs exert neuroprotective effects in a model of PD through the disassembly of aggregated α‐synuclein.

In the present study, MALDI‐TOF/MS analysis indicated that MMP‐2 is one of the MSC‐derived soluble factors that is involved in proteolysis of aggregated α‐synuclein and its related neurotrophic properties. MMPs are a family of zinc‐dependent endopeptidases that are functionally important for the extracellular space by degrading components of the extracellular matrix and basement membranes in disease processes [49]. The MMP genes are transcriptionally responsive to a wide variety of growth factors, cytokines, and reactive oxygen species [50, 51]. Recently, accumulating studies showed that MMPs are major culprits in the pathogenesis of PD mediated by neuroinflammation, apoptotic signaling in dopaminergic neurons, blood‐brain barrier disruption, and proteolysis of α‐synuclein aggregates [52, 53]. With regard to proteolytic effects of MMP on aggregated α‐synuclein, MMP‐1 and ‐3 have been shown to enhance α‐synuclein aggregation via increased formation of the aggregation‐prone α‐synuclein domain resulting from limited proteolysis [54, 55]. Interestingly, we demonstrated in the present study that the MSC‐derived factor, MMP‐2, could cleave preformed fibrils of α‐synuclein and then induce a compositional change of α‐synuclein fibrils to a smaller proportion of insoluble and oligomeric forms. This modulatory effect of MMP‐2 on aggregated α‐synuclein was further supported by MMP‐2 siRNA treatment in vitro, showing that MMP‐2 siRNA treatment of MSC‐CM resulted in loss of its proteolytic effects on aggregated α‐synuclein and its associated prosurvival effects on neuronal cells. Furthermore, our in vivo data clearly demonstrated that MMP‐2 was coexpressed within MSCs injected intravenously, and MSC treatment significantly increased the expression of MMP‐2 in MPTP‐treated and α‐synuclein‐inoculated animals, which finally attenuated the expression of α‐synuclein and the induction of pathogenic α‐synuclein. In animals treated with enriched α‐synuclein, this modulatory effect of MMP‐2 on aggregated α‐synuclein seemed to be comparable to those of MSCs, by showing that MMP‐2 treatment markedly decreased the extent of inoculated α‐synuclein aggregates, as well as expression of the insoluble and oligomeric forms of α‐synuclein, compared with fresh medium.

In agreement with our findings, a previous postmortem study indicated that the levels of MMP‐2 were significantly decreased in the substantia nigra of PD patients compared with age‐matched controls [56]. In addition, MMP‐2 appears to play an important role in degrading Alzheimer disease (AD) pathologies of Aβ40 and Aβ42 in cellular and animal models [57, 58]. Mlekusch and Humpel [59] suggested that a decrease in the level of MMP‐2 in cerebrospinal fluid of AD patients might contribute to the accumulation of insoluble Aβ peptide in plaques. Accordingly, the present study provided convincing evidence that MMP‐2, as one MSC‐derived soluble factor, can modulate the pathogenic microenvironments of PD through proteolysis of aggregated α‐synuclein into soluble forms.

Even though MMP‐2 had the potential to facilitate proteolysis against aggregated α‐synuclein, the ability of MMP‐2 was not comparable to that of MSCs. According to our in vitro data, the modulatory effect of MSCs on aggregated α‐synuclein and their related prosurvival effect on neuronal cells were not completely blocked by MMP‐2 siRNA treatment of CM, suggesting the existence of other MSC‐derived soluble factors involved in aggregated α‐synuclein cleavage. For example, several candidate proteases have been shown to cleave and degrade α‐synuclein, including neurosin, cathepsin D, and plasmin [60, 61, 62]. In addition, several neurotrophic factors secreted from MSCs would act as strong modulators of neuronal cell survival and neuroprotection against the PD‐related microenvironment [63]. Additional studies are therefore required to identify MSC‐derived small molecules responsible for α‐synuclein cleavage that would have clinical potential for the development of disease‐modifying therapeutic strategies for use in PD.

Because clinical and experimental data convincingly suggest a crucial role for α‐synuclein aggregation in the pathogenesis of PD, therapeutic strategies focusing on the disassembly of α‐synuclein aggregation have been investigated in both preclinical and clinical fields [64, 65]. Strategies that intervene with toxic protein aggregation can be divided into seed clearance, inhibition of aggregation formation, and aggregate clearance. Of these, the present data provide the role of MSCs in aggregate clearance, concomitantly showing that direct disassembly of extracellular α‐synuclein aggregation does not lead to increases in the levels of toxic aggregates. Considering our previous data indicating that, in addition to their advantages in clinical applications, MSCs have a modulatory role on intracellular α‐synuclein aggregation through autophagy [21], a therapeutic perspective suggests that the use of MSCs or MSC‐derived soluble factors as pharmacological modulators of α‐synuclein aggregation may be an effective therapeutic approach for PD.

## Conclusion

The present data indicated that MSCs exert neuroprotective properties through proteolysis of aggregated α‐synuclein into soluble forms, and MMP‐2 may be the principal soluble factor released by MSCs responsible for proteolysis of α‐synuclein aggregates. The present study suggests that the disassembly of α‐synuclein aggregation to control PD‐related microenvironments using MSCs may have a significant impact on future PD treatment strategies.

## Author Contributions

S.H.O.: conception and design, collection and/or assembly of data, manuscript writing, final approval of manuscript; H.N.K., H.J.P., J.Y.S., and D.Y.K.: technical assistance, final approval of manuscript; P.H.L.: supervision of study, data analysis and interpretation, financial support, final approval of manuscript.

## Disclosure of Potential Conflicts of Interest

The authors indicated no potential conflicts of interest.

## Supporting information

Supporting Information
