# Supplementary material for: The Cleavage Effect of Mesenchymal Stem Cell and Its Derived Matrix Metalloproteinase‐2 on Extracellular α‐Synuclein Aggregates in Parkinsonian Models
Source: Stem Cells Transl Med. 2016 Oct 11;6(3):949–61. doi: 10.5966/sctm.2016-0111 (PMC5442774; doi:10.5966/sctm.2016-0111)
Supplement: Supplementary file 1 — Supporting Information [file SCT3-6-0949-s001.pdf]

Supplemental Information

The cleavage effect of mesenchymal stem cell and its derived matrix metalloproteinase-2 on extracellular α-synuclein aggregates in parkinsonian models

Se Hee Oh, Ha Na Kim, Hyun Jung Park, Jin Young Shin, Dong Yeol Kim, Phil Hyu Lee

MMP-2 protein

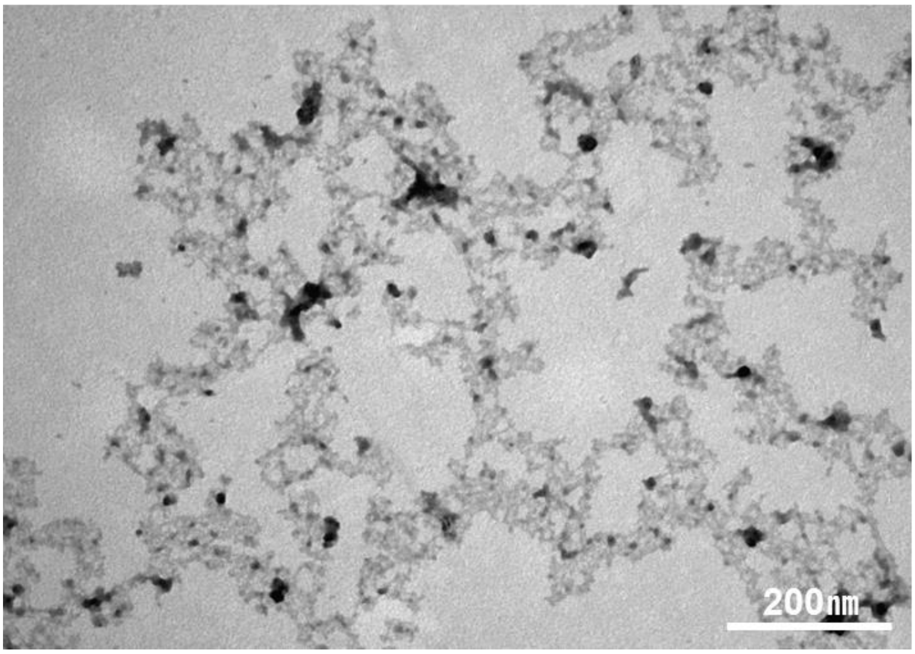

Supplemental Fig. 1. Morphological characteristic of recombinant MMP-2 protein by electron microscopy.

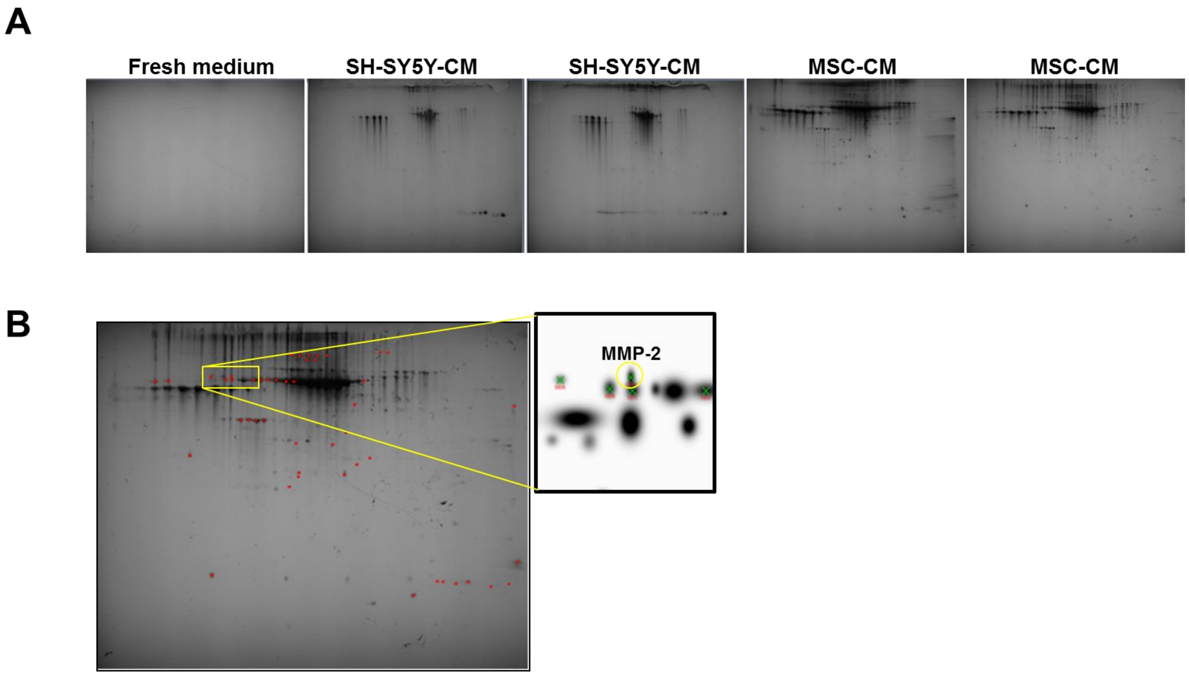

Supplemental Fig. 2. 2D-PAGE and MALDI-TOF/MS proteomics approaches from MSC-CM. (A) On the 2D-PAGE gel of total protein extracted from each medium at 72 hr, spot intensities and patterns were largely similar between independent samples from the SH-SY5Y-CM group and MSC-CM group. Fresh medium contained 6 discernable polypeptides, SH-SY5Y-CM contained 87 and 96 discernable polypeptides, and MSC-CM contained 189 and 157 discernable polypeptides by silver staining. (B) The analysis identified 47 spots in MSC-CM that significantly differed by at least twofold in expression level compared with fresh or SH-SY5Y-CM. The area shown in the yellow box in the left panel is enlarged in the right panels. MMP-2 protein was one of them expressed by yellow circle in enlarged image.

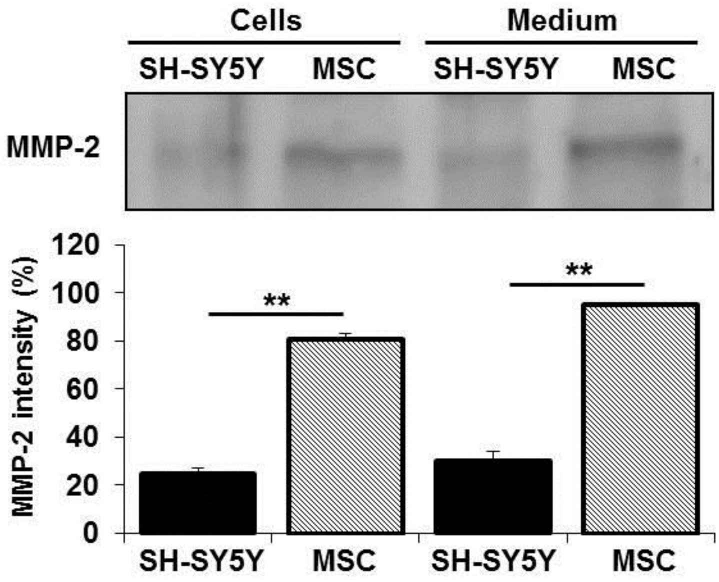

Supplemental Fig. 3. The expression of MMP-2 in MSCs and SH-SY5Y cells. MMP-2 was expressed in both MSCs and SH-SY5Y cells; however, the expression level of MMP-2 was much higher, approximately 4 times higher relative to SH-SY5Y cells. $^{**}P < 0.01$.

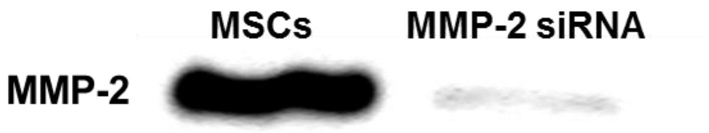

Supplemental Fig. 4. RT-PCR analysis of MMP-2 in MSCs. Transfection of MSCs with a MMP-2 siRNA construct effectively downregulated endogenous expression of MMP-2 mRNA.

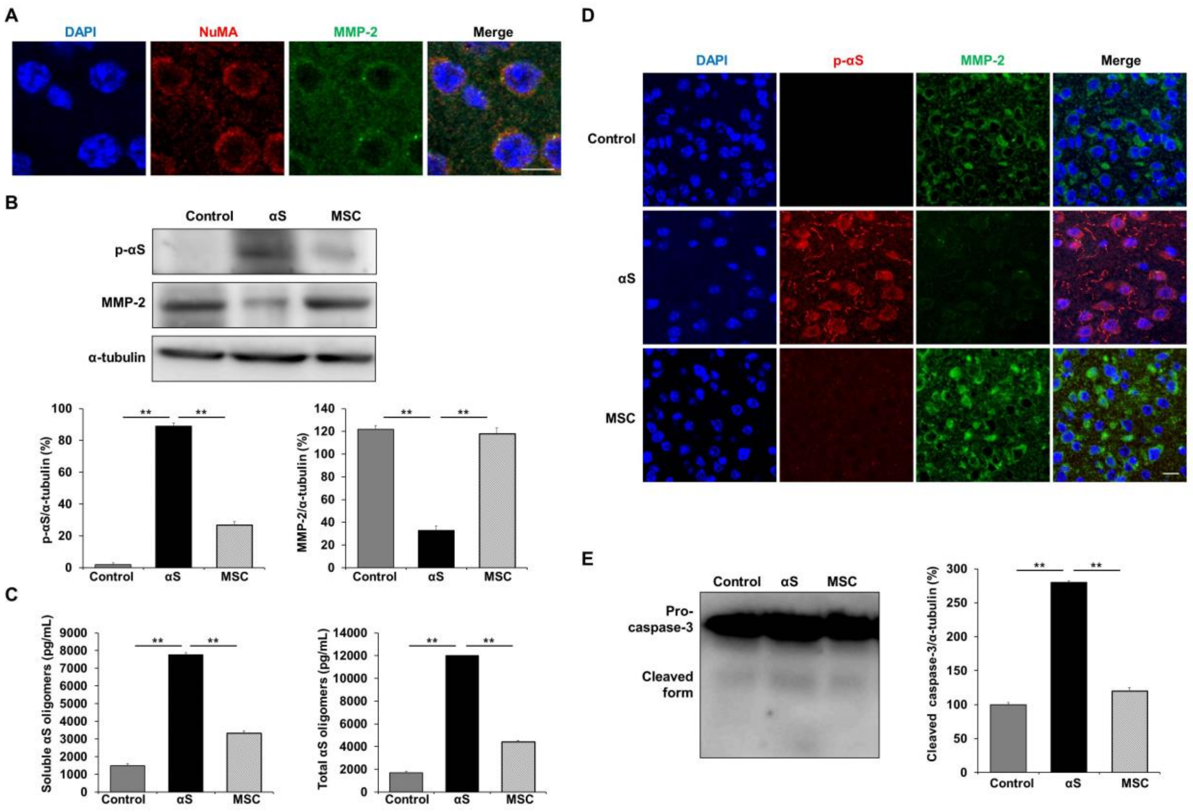

Supplemental Fig. 5. The effects of MSCs on proteolysis of α-synuclein aggregates following stereotaxic inoculation of α-synuclein fibrils into the striatum. (A, B) In α-synuclein-inoculated animals, NuMA-positive cells were co-immunostained with MMP-2 in the MSC-treated animal, and the expression of MMP-2 in the striatum was significantly increased in the MSC-treated animals relative to only α-synuclein-inoculated animals. (B, C) MSC administration in α-synuclein-inoculated animals significantly attenuated the amount of phosphorylated α-synuclein and the levels of total and soluble α-synuclein oligomers in the striatum compared to only α-synuclein-inoculated animals. (D) MSC treatment in α-synuclein-inoculated animals significantly attenuated phosphorylated α-synuclein immunoreactivity with an increase in MMP-2 immunoreactivity. (E) MSC treatment in α-synuclein-inoculated animals significantly decreased the expression of cleaved caspase-3 fragment in the striatum compared to only α-synuclein-inoculated animals. ***P* < 0.01. Scale bars, 10 μm.

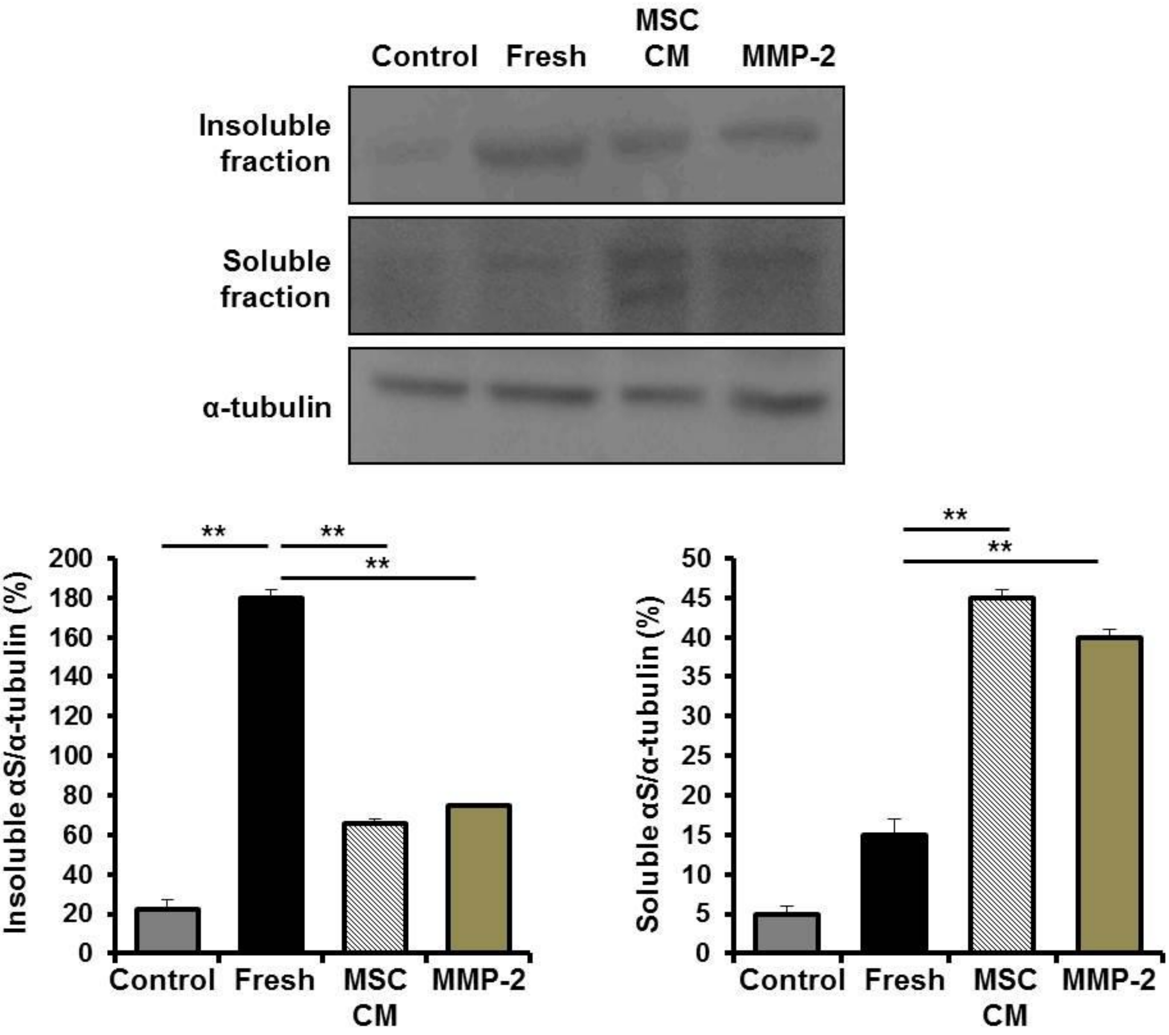

Supplemental Fig. 6. Western blotting using human specific α-synuclein antibody to uncover more precisely the amount of inoculated α-synuclein. Western blotting showed that the amount of inoculated insoluble α-synuclein was increased in animals receiving fresh medium compared to animals receiving MSC-CM or MMP-2; however, the amount of soluble α-synuclein was decreased in animals receiving fresh medium compared to animals receiving MSC-CM or MMP-2. $^{**}P < 0.01$.
